# Smartphone and wearable detected atrial arrhythmias in Older Adults: Results of a fully digital European Case finding study

**DOI:** 10.1093/ehjdh/ztac067

**Published:** 2022-11-01

**Authors:** L Fabritz, D L Connolly, E Czarnecki, D Dudek, E Guasch, D Haase, T Huebner, A Zlahoda-Huzior, K Jolly, P Kirchhof, J Obergassel, U Schotten, E Vettorazzi, S J Winkelmann, A Zapf, R B Schnabel, Smart in OAC—AFNET 9 investigators

**Affiliations:** University Center of Cardiovascular Science, University Heart and Vascular Center Hamburg, University Medical Center Hamburg-Eppendorf, Martinistr. 52, 20251 Hamburg, Germany; Department of Cardiology, University Heart and Vascular Center Hamburg, Martinistr. 52, 20251 Hamburg, Germany; DZHK German Center for Cardiovascular Research, partner site Hamburg/Luebeck/Kiel, Germany; Institute of Cardiovascular Sciences, University of Birmingham, Edgbaston Wolfson Drive, B15 2TT Birmingham, UK; Atrial Fibrillation NETwork (AFNET), Mendelstr 11, 48149 Münster, Germany; Institute of Cardiovascular Sciences, University of Birmingham, Edgbaston Wolfson Drive, B15 2TT Birmingham, UK; Department of Cardiology and R&D, Birmingham City Hospital, Sandwell and West Birmingham Trust, Dudley Road, B18 7QH Birmingham, UK; Atrial Fibrillation NETwork (AFNET), Mendelstr 11, 48149 Münster, Germany; Jagiellonian University Medical College, Center for Digital Medicine and Robotics, Ul. Kopernika 7E, 33-332 Kraków, Poland; Maria Cecilia Hospital, Via Corriera, 1, 48033 Cotignola RA, Italy; Institut Clínic Cardio-Vascular, Hospital Clínic, University of Barcelona, Carrer de Villaroel, 170, 08036 Barcelona, CA, Spain, Spain; IDIBAPS, Rosselló 149-153, 08036 Barcelona, CA, Spain; CIBERCV, Monforte de Lemos 3-5, Pabellon 11, Planta 0, 28029 Madrid, Spain; Atrial Fibrillation NETwork (AFNET), Mendelstr 11, 48149 Münster, Germany; Preventicus GmbH, Ernst-Abbe-Straße 15, 07743 Jena, Germany; Department of Measurement and Electronics, AGH University of Science and Technology, Al. Mickiewicza 30, 30-059 Kraków, Poland; Institute of Applied Health Research, University of Birmingham, Edgbaston, B15 2TT Birmingham, UK; Department of Cardiology, University Heart and Vascular Center Hamburg, Martinistr. 52, 20251 Hamburg, Germany; DZHK German Center for Cardiovascular Research, partner site Hamburg/Luebeck/Kiel, Germany; Institute of Cardiovascular Sciences, University of Birmingham, Edgbaston Wolfson Drive, B15 2TT Birmingham, UK; Atrial Fibrillation NETwork (AFNET), Mendelstr 11, 48149 Münster, Germany; University Center of Cardiovascular Science, University Heart and Vascular Center Hamburg, University Medical Center Hamburg-Eppendorf, Martinistr. 52, 20251 Hamburg, Germany; Department of Cardiology, University Heart and Vascular Center Hamburg, Martinistr. 52, 20251 Hamburg, Germany; DZHK German Center for Cardiovascular Research, partner site Hamburg/Luebeck/Kiel, Germany; Atrial Fibrillation NETwork (AFNET), Mendelstr 11, 48149 Münster, Germany; Department of Physiology, Cardiovascular Research Institute Maastricht, Maastricht University Medical Center +, Debyelaan 25, 6229 HX, Maastricht, The Netherlands; Institute of Medical Biometry and Epidemiology, University Medical Center Hamburg-Eppendorf, Christoph-Probst-Weg 1, 20246 Hamburg, Germany; University Center of Cardiovascular Science, University Heart and Vascular Center Hamburg, University Medical Center Hamburg-Eppendorf, Martinistr. 52, 20251 Hamburg, Germany; Department of Cardiology, University Heart and Vascular Center Hamburg, Martinistr. 52, 20251 Hamburg, Germany; Institute of Medical Biometry and Epidemiology, University Medical Center Hamburg-Eppendorf, Christoph-Probst-Weg 1, 20246 Hamburg, Germany; Department of Cardiology, University Heart and Vascular Center Hamburg, Martinistr. 52, 20251 Hamburg, Germany; DZHK German Center for Cardiovascular Research, partner site Hamburg/Luebeck/Kiel, Germany; Atrial Fibrillation NETwork (AFNET), Mendelstr 11, 48149 Münster, Germany

**Keywords:** Atrial arrhythmia, Consumer electronics, Elderly population, Early detection, Digital trial

## Abstract

**Aims:**

Simplified detection of atrial arrhythmias via consumer-electronics would enable earlier therapy in at-risk populations. Whether this is feasible and effective in older populations is not known.

**Methods and results:**

The fully remote, investigator-initiated **Smart**phone and wearable detected atrial arrhythmia **in O**lder **A**dults **C**ase finding study (Smart in OAC—AFNET 9) digitally enrolled participants ≥65 years without known atrial fibrillation, not receiving oral anticoagulation in Germany, Poland, and Spain for 8 weeks. Participants were invited by media communications and direct contacts. Study procedures adhered to European data protection. Consenting participants received a wristband with a photoplethysmography sensor to be coupled to their smartphone. The primary outcome was the detection of atrial arrhythmias lasting 6 min or longer in the first 4 weeks of monitoring. Eight hundred and eighty-two older persons (age 71 ± 5 years, range 65–90, 500 (57%) women, 414 (47%) hypertension, and 97 (11%) diabetes) recorded signals. Most participants (72%) responded to adverts or word of mouth, leaflets (11%) or general practitioners (9%). Participation was completely remote in 469/882 persons (53%). During the first 4 weeks, participants transmitted PPG signals for 533/696 h (77% of the maximum possible time). Atrial arrhythmias were detected in 44 participants (5%) within 28 days, and in 53 (6%) within 8 weeks. Detection was highest in the first monitoring week [incidence rates: 1st week: 3.4% (95% confidence interval 2.4–4.9); 2nd–4th week: 0.55% (0.33–0.93)].

**Conclusion:**

Remote, digitally supported consumer-electronics-based screening is feasible in older European adults and identifies atrial arrhythmias in 5% of participants within 4 weeks of monitoring (NCT04579159).

## Graphical Abstract

**Graphical Abstract:**
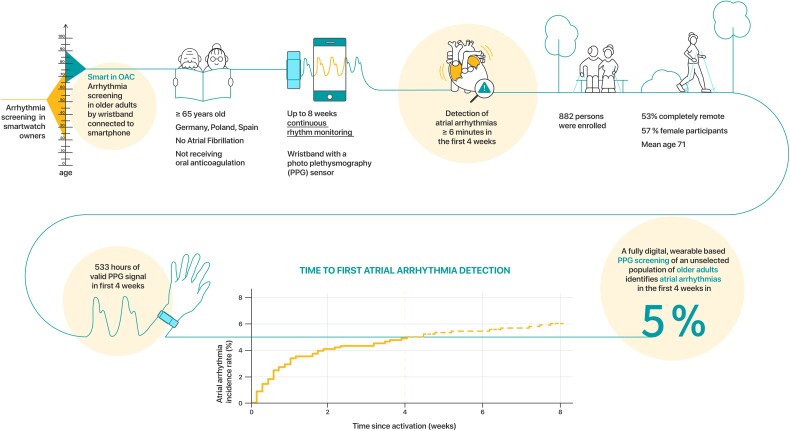

## Introduction

Earlier initiation of anticoagulation could prevent strokes and cardiovascular deaths in patients with atrial fibrillation (AF).^1–4^ Recently controlled clinical trials demonstrate that population-based screening for AF and subsequent initiation of oral anticoagulation can prevent some strokes.^5,6^ These trials led to recent recommendations in a practical guide of the European Heart Rhythm Association (EHRA) to intermittently screen individuals aged ≥ 75 years and consider systematic screening in individuals aged ≥ 65 years with additional comorbidities contributing to stroke risk.^7^ However, these studies also illustrate relatively high numbers needed to screen. Recent trials have shown that patient-operated ECG monitors can be rolled out to preselected screening populations.^5^ Implanted cardiac monitors are associated with a high analysable monitoring time,^6^ but involve invasive procedures. Simple, scalable methods to identify atrial arrhythmias in at-risk populations are needed to enable the timely detection of AF and initiation of therapy.

Continuous rhythm screening using implanted pacemakers or ECG monitors detects short atrial arrhythmias in up to 30% of elderly participants,^6,8^ but is limited by its invasive nature. Atrial arrhythmias that are only detected during many months of monitoring must statistically occur less often or be of shorter durations than arrhythmias that occur more often and are longer and therefore are more likely to be detected in shorter monitoring periods.^6,9,10^ Indeed, subclinical AF detected in implantable cardiac devices is associated with a lower stroke risk than clinical AF,^9–11^ although a cut-off point for increased stroke risk remains to be found and validated.^12–14^ Modern consumer electronics, including smartphones and smartwatches or wearable-based devices,^15–17^ enable recording of pulse plethysmography (PPG). Combined with validated analysis algorithms,^18,19^ this can be applied to monitor for arrhythmias.^19^ Wearable-based screening for atrial arrhythmias is feasible when company-owned data are analysed in relatively young, early adopters.^15–17,19,20^ An analysis of previously reported atrial arrhythmia detection rates with wearables is summarized in *Table 2* and Supplementary material online, *Figure S1*. The US Screening Task Force and an EHRA practical guide recognized the potential of PPG-based arrhythmia screening,^7^ but noted that more evidence was needed before it could be recommended,^1^ especially regarding arrhythmia screening in older populations.^1,5,6^ Inclusive methods offering PPG-based arrhythmia screening to older participants are therefore required.^7^

To address this societal need, the **Smart**phone and wearable detected atrial arrhythmia **in O**lder **A**dults **C**ase finding study (Smart in OAC—AFNET 9) evaluated the usability of a fully digital, PPG-based detection system for atrial arrhythmias in older European adults.^21^

## Methods

### Study design

Smart in OAC—AFNET 9 is an investigator-initiated, single-arm, international, multicentre case-finding study in an at-risk population without previously known AF using a low-threshold, digitally enhanced screening platform (https://clinicaltrials.gov/ct2/show/NCT04579159). Details of the study design have been published.^21^ The study has been approved by the local Ethics Committees in all participating sites [Hamburg 2020–10260-BO-ff, Dresden (Markkleeberg) EK-BR-95/21–1, Barcelona HCB/2021/0255, Krakow/Nowy Sasz 298/KBL/OIL/2020, Birmingham, UK IRAS 292218]. To capture societal and health care realities in different parts of Europe, the study was planned in Germany (Central Europe), Poland (Eastern Europe), Spain (Western Europe), and the UK (central NHS system). In the UK, administrative delays due to COVID-19 prevented the study from commencement in time. Sponsor of the trial is AFNET (https://www.kompetenznetz-vorhofflimmern.de). Financial support came from Daiichi-Sankyo Europe in the form of an unrestricted grant and by Preventicus, Jena, Germany, as an in-kind contribution.

### Participants

Potential participants aged 65 years or older without known AF and not on oral anticoagulation were made aware of the study using newspaper and television advertisements targeting audiences of older adults, senior citizen interest groups, personal contacts in the sites, general physicians in the community, leaflets, and a website.

*Study intervention.* Within the limitations of a case finding study requiring consent, the system was designed for simplicity. After expressing interest and agreeing to be contacted using digital, oral, or written communication, potential participants were offered participation. Informed consent was obtained digitally. Paper versions were available on demand and were required in Spain. A wristband with a PPG sensor (Corsano 287, MMT SA, Switzerland) was shipped to consenting participants or collected at the site. Participants installed the Corsano Preventicus Smart app onto their smartphone (operating system requirements Apple iOS version 12.2 or higher or Android 8.0 or higher) and coupled the wristband via Bluetooth for app-transferal of PPG data. The wearable technology records and transfers passively around the clock, operating for up to 5 days between recharging. Participants were asked to wear the wristband and use the system for 4 weeks with the possibility to extend monitoring for up to 8 weeks if atrial arrhythmias had not been found. Analysis of the pulse waves for atrial arrhythmias used a validated algorithm (Class IIa CE certified medical product, Preventicus Heartbeats®, Jena, Germany, www.preventicus.com,^18,19^). All signals were centrally analysed by a cloud-based and device-agnostic analytic service (Preventicus Heartbeats, CE marked certified medical device.^21^) Although the Corsano wristband was used and the app was adapted to Corsano technology, any other high**-**quality PPG wristband could be used in the future.

The PPG was continuously recorded with the wristband and split in 1 min-long segments, each of them analysed via the atrial arrhythmia detection algorithm. One-minute recordings were excluded automatically if more than 10% of the signal had poor quality, e.g. from movement artefacts. Length of atrial arrhythmia episodes was estimated via consecutive positive one-minute segments and atrial arrhythmias in this study were defined as periods of an irregular PPG signal lasting six minutes or longer or a burden of 1.5% per 24 h or more.^21^ Atrial arrhythmias of this duration detected by implanted devices are associated with an increased risk of stroke.^10,22^ PPG analysis was stopped after the detection of atrial arrhythmias.

To ensure that participants would be reassured or receive a diagnosis of AF and subsequent treatment as required despite restrictions of health services during the pandemic, all participants with positive PPG atrial arrhythmia screening were offered a 14-day external loop recorder Holter ECG (CardioMem® CM 100 XT), delivered by post or handed out on site. The same loop recorder was also planned to be offered to a random sample of participants without PPG detection of atrial arrhythmias, while the delivery of Holter ECG recorders to positively PPG screened participants was prioritized.

### Data collection

Information on name, mobile number, date of birth, known AF, and current oral anticoagulation was entered by the participants via their smartphone at enrolment and during the screening process (*Table 1*). The results of the PPG analyses and the Holter ECG were captured on the systems described above and integrated into the final data set for analysis. The results of this investigation were made available to the site teams for medical action.

**Table 1:** Clinical characteristics of the study participants and of participants with and without atrial arrhythmia (AA)

	Total (*n* = 882)	without atrial arrhythmias (*n* = 838)	with atrial arrhythmias (*n* = 44)	*P*-value
**Age**				0.008^1^
Mean ± SD,	70.9 ± 4.9	70.8 ± 4.8	72.8 ± 5.7	
Median (Q1, Q3)	70.0 (67.0, 74.0)	69.0 (67.0, 74.0)	71.5 (68.8, 75.2)	
Range	65.0–90.0	65.0–90.0	65.0–86.0	
**Sex**				0.797^2^
female	500 (56.7%)	473 (56.4%)	27 (61.4%)	
male	381 (43.2%)	364 (43.4%)	17 (38.6%)	
other	1 (0.1%)	1 (0.1%)	0 (0.0%)	
**Country**				0.914^2^
Germany	575 (65.2%)	546 (65.2%)	29 (65.9%)	
Poland	277 (31.4%)	263 (31.4%)	14 (31.8%)	
Spain	30 (3.4%)	29 (3.5%)	1 (2.3%)	
**data source**				0.963^2^
GP	80 (9.1%)	76 (9.1%)	4 (9.1%)	
Hospital	20 (2.3%)	19 (2.3%)	1 (2.3%)	
Leaflet	96 (10.9%)	92 (11.0%)	4 (9.1%)	
Other	633 (71.8%)	600 (71.6%)	33 (75.0%)	
Pharmacy	15 (1.7%)	15 (1.8%)	0 (0.0%)	
Website	38 (4.3%)	36 (4.3%)	2 (4.5%)	
**Measurement bracelet received**				0.619^2^
Post	469 (53.2%)	444 (53.0%)	25 (56.8%)	
Site	413 (46.8%)	394 (47.0%)	19 (43.2%)	
**Ethnic origin**				0.965^2^
Arab	2/851 (0.2%)	2/808 (0.2%)	0/43 (0.0%)	
Asian	2/851 (0.2%)	2/808 (0.2%)	0/43 (0.0%)	
Mixed	2/851 (0.2%)	2/808 (0.2%)	0/43 (0.0%)	
Other	62/851 (7.3%)	58/808 (7.2%)	4/43 (9.3%)	
White	783/851 (92.0%)	744/808 (92.1%)	39/43 (90.7%)	
**Hypertension**				0.543^2^
No	439/853 (51.5%)	416/812 (51.2%)	23/41 (56.1%)	
Yes	414/853 (48.5%)	396/812 (48.8%)	18/41 (43.9%)	
**Diabetes mellitus**				0.057^2^
No	762/859 (88.7%)	720/816 (88.2%)	42/43 (97.7%)	
Yes	97/859 (11.3%)	96/816 (11.8%)	1/43 (2.3%)	
**EQ-5D: mobility**				0.522^3^
Nmiss	331	313	18	
I have no problems in walking about	471 (85.5%)	449 (85.5%)	22 (84.6%)	
I have slight problems in walking about	51 (9.3%)	50 (9.5%)	1 (3.8%)	
I have moderate problems in walking about	26 (4.7%)	23 (4.4%)	3 (11.5%)	
I have severe problems in walking about	3 (0.5%)	3 (0.6%)	0 (0.0%)	
I am unable to walk about	0 (0.0%)	0 (0.0%)	0 (0.0%)	
**EQ-5D: self-care**				0.158^3^
Nmiss	331	313	18	
I have no problems washing or dressing myself	538 (97.6%)	513 (97.7%)	25 (96.2%)	
I have slight problems washing or dressing myself	11 (2.0%)	11 (2.1%)	0 (0.0%)	
I have moderate problems washing or dressing myself	2 (0.4%)	1 (0.2%)	1 (3.8%)	
I have severe problems washing or dressing myself	0 (0.0%)	0 (0.0%)	0 (0.0%)	
I am unable to wash or dress myself	0 (0.0%)	0 (0.0%)	0 (0.0%)	
**EQ-5D: usual activities**				0.666^3^
Nmiss	330	312	18	
I have no problems doing my usual activities	498 (90.2%)	475 (90.3%)	23 (88.5%)	
I have slight problems doing my usual activities	41 (7.4%)	39 (7.4%)	2 (7.7%)	
I have moderate problems doing my usual activities	13 (2.4%)	12 (2.3%)	1 (3.8%)	
I have severe problems doing my usual activities	0 (0.0%)	0 (0.0%)	0 (0.0%)	
I am unable to do my usual activities	0 (0.0%)	0 (0.0%)	0 (0.0%)	
**EQ-5D: Pain and discomfort**				0.509^3^
Nmiss	334	316	18	
I have no pain or discomfort	316 (57.7%)	300 (57.5%)	16 (61.5%)	
I have slight pain or discomfort	172 (31.4%)	164 (31.4%)	8 (30.8%)	
I have moderate pain or discomfort	48 (8.8%)	46 (8.8%)	2 (7.7%)	
I have severe pain or discomfort	11 (2.0%)	11 (2.1%)	0 (0.0%)	
I have extreme pain or discomfort	1 (0.2%)	1 (0.2%)	0 (0.0%)	
**EQ-5D: anxiety and depression**				0.232^3^
Nmiss	329	311	18	
I am not anxious or depressed	447 (80.8%)	423 (80.3%)	24 (92.3%)	
I am slightly anxious or depressed	83 (15.0%)	82 (15.6%)	1 (3.8%)	
I am moderately anxious or depressed	21 (3.8%)	20 (3.8%)	1 (3.8%)	
I am severely anxious or depressed	1 (0.2%)	1 (0.2%)	0 (0.0%)	
I am extremely anxious or depressed	1 (0.2%)	1 (0.2%)	0 (0.0%)	
**EQ-5D VAS**				0.454^1^
Nmiss	331	313	18	
Mean ± SD	82.9 ± 12.8	83.0 ± 12.6	81.1 ± 17.6	
Median (Q1, Q3)	85.0 (80.0, 91.0)	85.0 (80.0, 91.0)	86.0 (80.0, 90.0)	
Range	20.0–100.0	29.0–100.0	20.0–100.0	
**EQ-5D 5L VT score**				0.566^1^
Nmiss	339	321	18	
Mean ± SD	0.95 ± 0.08	0.95 ± 0.09	0.96 ± 0.06	
Median (Q1, Q3)	0.97 (0.92, 1.00)	0.97 (0.92, 1.00)	1.00 (0.94, 1.00)	
Range	0.28–1.00	0.28–1.00	0.80–1.00	

Baseline characteristics of the study population grouped by AA detection within the first 28 days. Categorical data are *n* (%) or *n*/valid *n* (%) in case of missing values. Age is presented as mean ± SD, EQ-5D VAS, and EQ-5D 5L VT Score as median (IQR). EQ-5D VAS was missing for total *n* = 331, without AA *n* = 313, with AA *n* = 18, EQ-5D 5L VT Score was missing for *n* = 339/321/18. (1) Linear Model ANOVA, (2) Pearson’s χ^2^ test, (3) Trend test for ordinal variables.

(1) Student’s *t*-test, (2) Pearson’s χ^2^, (3) Armitage trend test for ordinal variables, Nmiss, number of missing values; GP, general practitioner.

Preventicus data management and data protection comply with General Data Protection Regulations. Personal data (declarations of consent, contact information, etc.) were stored exclusively in a defined cloud workspace (Preventicus Caresafe). The data in the Caresafe were end-to-end encrypted, limiting access to personal data to study site staff. Preventicus did not have any access to the personal data of participants.

### Statistical considerations

#### Sample size

AA are detected in circa 30–40% of elderly populations when continuous monitoring is applied for 2–3 years using implantable loop recorders.^6,8,23^ Integrating the estimated effects of shorter monitoring times (1 month), considering that the wearable will not record continuously due to noise and the need for charging, and based on the known effects of intermittent and shorter ECG monitoring on detection rates of short AA,^9,17,24^ we assumed a detection rate of AA of 3–6% in the screening population.^21^ A sample size of 1000 participants would allow us to estimate a rate of detection of 5% with a precision of 2.8% (width of the two-sided 95% Clopper-Pearson confidence interval (CI), PASS 16.0.3), a sample size of 750 gives a precision of 3.3%.

#### Primary outcome

The primary outcome parameter of this study is the prevalence of PPG-detected atrial arrhythmias (lasting six minutes or longer), calculated as the number of participants with AA detected by the wearable in relation to all included participants. The primary analysis assessed atrial arrhythmias detected in 4 weeks of monitoring.

#### Secondary outcomes

Secondary outcomes include the total number of participants with atrial arrhythmias over the entire 8-week recording; time from enrolment to AA detection with death as a competing risk; regional differences in AA detection and differences by route of invitation; quality of life estimated by EQ-5D-5L in participants with and without AA; detection of AF by ECG, compliance, and reasons for non-participation.

#### Adverse events

SMART in OAC—AFNET 9 is a low-risk study using approved procedures to screen for atrial arrhythmias. Adverse events of interest related to the study procedures (e.g. unwanted effects of the wearable, in this case, a wristband) were noted by study centres if voiced by participants and collected in a questionnaire for participants following an invitation to a Holter ECG.

#### Statistical analyses

All analyses were prespecified in a dedicated statistical analysis plan signed on 31 January 2022 before accessing the data. The primary analysis was based on the full analysis data set (FAS), consisting of all participants that consented to screening and provided at least one data point. A sensitivity analysis was performed in a per protocol population including all participants that used the wearable as intended, i.e. used the device until screening rendered a positive result or in whom an analysable PPG signal was available for at least 300 h in the first 4 weeks of monitoring. Demographics and baseline characteristics are summarized using descriptive statistics. The detection rate of AA was calculated together with the corresponding two-sided 95% Clopper-Pearson CI. If participants discontinued participation, the information gathered until discontinuation was analysed. Time to first AA detection was analysed by taking death as a competing risk into account using Aalen-Johansen curves. A multivariable logistic model utilizing Firth’s bias-reduced penalized-likelihood was used to simultaneously identify predictors of AA. All analyses were carried out using R v4.0.5 (R Core Team, Vienna).

#### Role of the funding source

Smart in OAC—AFNET 9 is an investigator-initiated trial designed and executed by the authors. AFNET oversaw the trial as the legal sponsor, with U.S. serving as sponsor representative on the steering committee. Daiichi-Sankyo Europe provided funding for the study to AFNET and held a non-voting seat on the steering committee. Preventicus provided access to their PPG-based AF screening technology and Telecare Health system.

#### Data sharing

The protocol, informed consent in its written form, and statistical analysis plan are available in this paper. Study data will be made available for research purposes for at least 5 years after the completion of the study. Please direct inquiries including an outline of the planned analyses to info@kompetenznetz-vorhofflimmern.de; info@af-net.eu. Data will be made available by AFNET on reasonable request.

## Results

### Participants

**Figure 1:**
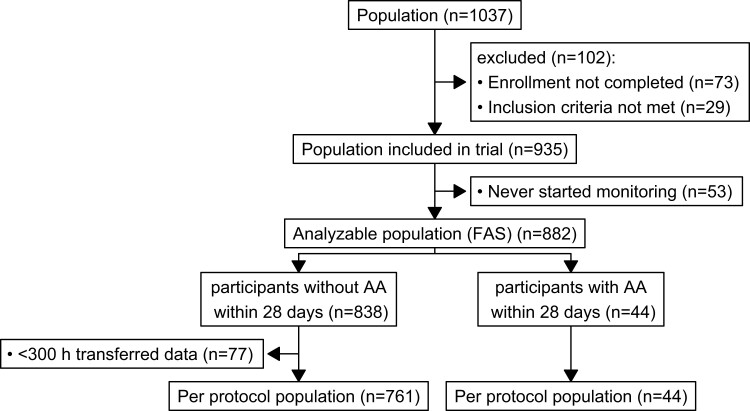
STROBE flow chart of the study. FAS, full analysis sample.

A total of 882 participants were PPG-screened in Germany, Poland, and Spain between 01 February 2021 and 31 January 2022 (*Figure 1*). In Spain, the Barcelona ethics committee required an in-person consent process with a hand-signed consent form. Five hundred (57%) participants were female, 414 (47%) participants reported known hypertension, and 97 (11%) reported known diabetes (*Table 1*). The age ranged from 65 to 90 years (mean age 71 ± 5 years). The majority of participants (72%) were reached by media campaigns in newspapers and television or by word of mouth and town hall meetings for senior citizens (category ‘other’ in Supplementary material online, *Figure S2*). The remaining participants were attracted by leaflets (11%), identified by general practitioners made aware of the study (9%), a website (4%), the site team hospital ambulatory settings (2%), or pharmacies (2%). Communication about the study in targeting audiences of older adults, including newspaper and television adverts, video messages, and town hall meetings, were associated with high recruitment rates (see Supplementary material online, *Figure S2*).

### Primary outcome

**Figure 2:**
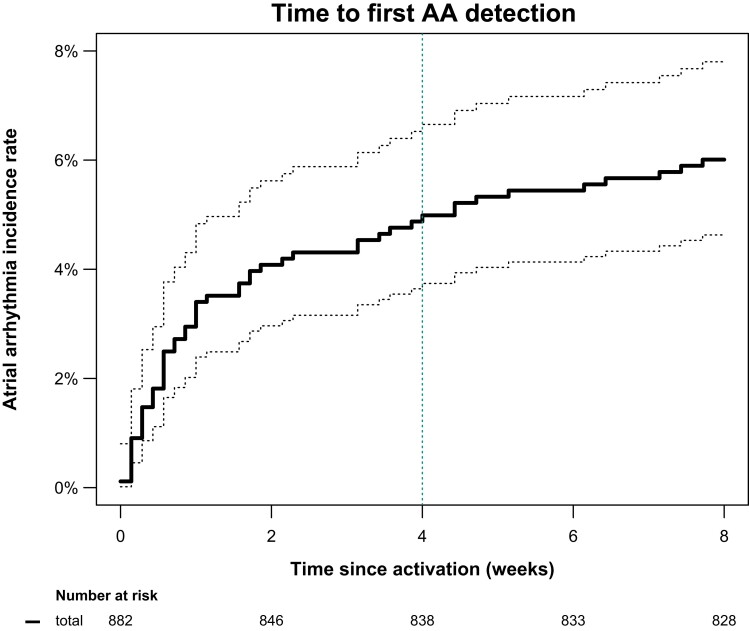
Detection of atrial arrhythmias over time in the study population. The bold continuous black line shows the Kaplan-Meier estimated cumulative event rate with corresponding 95% confidence interval (dotted black lines). The dotted green vertical line identifies the time point of the primary outcome, detection of atrial arrhythmias within 28 days of screening. Screening beyond this time point identified only a few additional cases.

Atrial arrhythmias were detected in 44/882 participants [5.0%, 95% CI (3.6–6.6)] within 28 days of monitoring (*Figure 2*). Arrhythmia detection rate was higher in the 1st week of monitoring compared with subsequent weeks: The atrial arrhythmia incidence rate was 3.4 participants with atrial arrhythmias/100 monitored weeks (95% CI 2.4–4.9) in the 1st week of monitoring and between 0.12 and 0.71 in subsequent weeks [average incidence rate for week 2–4 was 0.55 (0.33–0.93), *P* < 0.001 for incidence rate in the 1st week vs. weeks 2–4, *Figure 2*].

### Secondary outcomes

Atrial arrhythmias were detected in 53/882 participants (6%) within 8 weeks of monitoring (*Figure 2*). The time from initiation of monitoring to detection of atrial arrhythmias was relatively short, confirming the higher detection rate early during monitoring (*Figure 2*).

A prespecified sensitivity analysis confined to participants who used the device per protocol within the first 4 weeks of monitoring, found a similar detection rate of 44/805 5.5% (95% CI 4.0–7.3).

Participants with atrial arrhythmias were older than those without atrial arrhythmias (*Table 1* and *Figure 4*). There were no differences in the detection of atrial arrhythmias by region, by route of invitation to the study, or by sex (*Table 1* and *Figure 4*). Quality of life was similar in participants with atrial arrhythmias compared with those without atrial arrhythmias (*Table 1*). Older age was the only parameter associated with atrial arrhythmia detection in this study (*Figure 4*).

**Figure 3:**
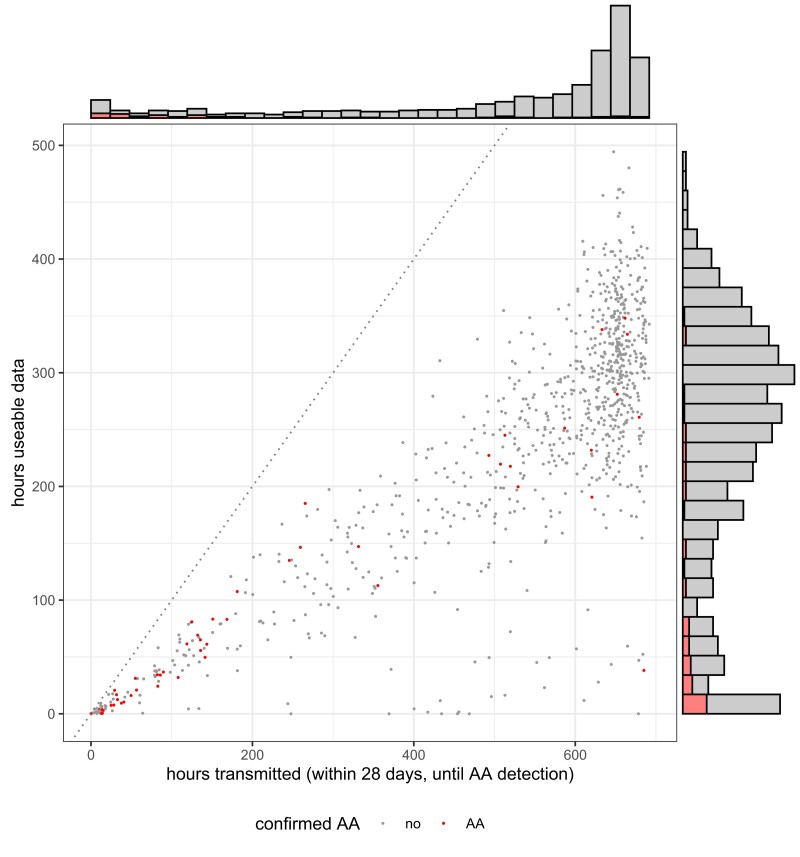
Distribution of wearable screening duration (hours transmitted on x-axis, hours usable on y-axis) PPG screening analysis stopped after AA was detected and confirmed by the analysis service.

**Figure 4:**
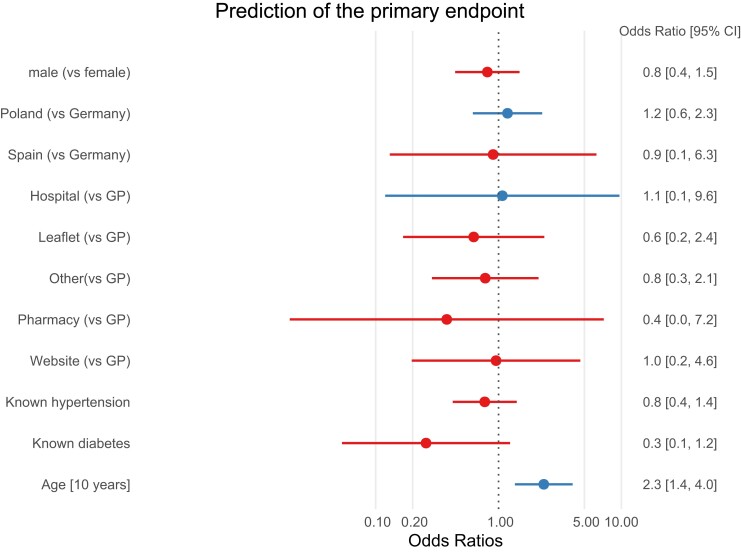
Forest plot of factors associated with AA. Older age was the only factor associated with atrial arrhythmias in this study.

Participants transmitted a mean of 530 h of PPG recordings over the first 696 h of monitoring (76% of the maximal monitoring duration within 4 weeks plus the inclusion day. Of these, 240 h (45%) were of sufficient quality for rhythm analyses (*Figure 3*). The transmission rate dropped slightly to 400 h/28 days in weeks 5–8 of monitoring.

Time of day of AA detection was evaluated in participants with any AA detection (53/882, 6%): There were no differences between the number of transmitted PPG-minutes observed between daytime (6:00 am to 10:00 pm) and nighttime. While 73% of recorded PPG-minutes during nighttime could be used for AA detection, only 26% of recorded PPG-minutes during daytime were analysable (*P* < 0.0001). In participants with any AA detection, AA burden at night was 1.57-fold higher than during the daytime, with daytime AA burden of 9 min/h and nighttime AA burden of 14 min/h of analysable recording [(95% CI 1.15–2.14), *P* = 0.004]. Just over half of the participants (53%) participated without any in-person contact, while 47% of participants received personal assistance with the device. At the Barcelona site, the 30 participants were required to sign a written informed consent. Technical problems with Bluetooth coupling and recoupling, omission of recharging the smartphone, or local skin irritation during the summer heat, were reasons for queries to technical support and study sites and discontinuation of monitoring. In addition to communication routes via the app, SMS, email, and staff at the study sites, a central technical telephone support hotline was provided. About half of the participants (51%) contacted the central telephone hotline for queries. Most of the queries regarded pairing and coupling for data transfer.

All 53 participants with PPG-detected atrial arrhythmias were invited to undergo a 14-day event recorder ECG. Of these, 45 later underwent a 14-day event recorder ECG as part of the study. Eight participants did not have that test as part of the study, as they either had symptoms and were diagnosed with AF in the hospital or aimed to receive further diagnostics elsewhere with results not known. An additional random control sample without PPG-detected atrial arrhythmias underwent the same event monitoring. Event monitoring started with a median delay of 31 days (IQR 21, 48) after AA detection in PPG. Event-recorder Holter ECGs identified AF in 27/45 participants with previously PPG-detected arrhythmias, and none (0/7) participants without PPG-detected arrhythmias.

Participants undergoing the study Holter ECG filled in a second questionnaire depicted in Supplementary material online, *Table S2*. The estimated CHA_2_DS_2_-VASc-Score was 2.6 ± 1.4, sufficient to decide on oral anticoagulation.

## Discussion

*Main findings.*  **Smart**phone and wearable detected atrial arrhythmia **in O**lder **A**dults **C**ase finding study (Smart in OAC—AFNET 9) successfully deployed a fully digital, consumer-electronics based system to detect atrial arrhythmias in older adults in several European countries. The main results are:

1. A fully digital wearable system worn for 4 weeks identifies atrial arrhythmias in 5% of older adults (> 65 years of age).
2. The majority of participants were identified using targeted public media communications or direct contacts. Offers of remote technical assistance were accepted and compliance was high, showing feasibility and scalability for this age group if targeted.
3. Detection rates for atrial arrhythmias are high in the 1st week of PPG monitoring, and taper off thereafter, suggesting that relatively short monitoring periods may be sufficient to detect older adults with atrial arrhythmias.

These findings encourage the use of fully digital, consumer-electronics based PPG systems to screen for atrial arrhythmias in older adults.

*Atrial arrhythmia detection in Smart in OAC—AFNET 9.* We systematically reviewed the performance of electronic devices to screen for atrial arrhythmias in older adults 65 years of age and above. Five systematic reviews of arrhythmia detection via mobile health applications published between 2020 and 2022^25–28^ yielded 28 potentially eligible studies. A MEDLINE search conducted on 25 February 2022 (search terms see Supplementary material online) revealed 235 unique records published between 2020 and 2022, containing 23 already found eligible via the initial five systematic reviews. During revision, the MEDLINE search was repeated and yielded additional 70 potentially eligible articles. Overall, 71 full texts were assessed which yielded 26 included studies (*Table 2*, Supplementary material online, *Figure S1*). In cases where age subgroup data were reported in a trial, it was still included and the incidence rate was calculated from the reported patient numbers.^15,16^ The same was performed when the original study only reported comparative outcomes such as hazard ratio but counts of diagnosed patients and totally screened patients were also reported.^37^

**Table 2:** Studies using different heart rate monitoring techniques to detect atrial arrhythmias in older adults with focus on wearables

Study	Monitoring	Technique	Author, study acronym *subgroup/own analysis*	Population	Design, intervention	Mean age (+/− Std.)	Female (%)	*n*	Atrial arrhythmia IR (%)
General population/community based/at risk cohorts, remote	Continuous monitoring	single lead ECG	Bowman/Casadei et al. AMALFI *From ISRCTN*	General population based Region: K Age: ≥ 64 Inclusion: CHA_2_DS_2_-VASc ≥ 4 in woman or ≥ 3 in men Exclusion: Known AF: excluded	Sequence: continuous, 14 days Biodata: single lead ECG Device: ZioPatch	Analysis ongoing/unpublished			
			Langer et al. 2021^29^ AWARE-AF program	Community based, recruited from outpatient cardiology Region: Canada Age: ≥ 65 Inclusion: ≥ 1 of poAF, ST/TIA or ≥ 75 years old + ≥ 2 of HF, HT, DM, CKD, OSAS, CVD, EF < 35%, significant DDys Exclusion: implanted PM/ICD Known AF: excluded	Sequence: continuous, 7 days Biodata: single lead ECG Device: Cardiostat, Canada	78.7 ± 6.1	42%	942	2.7 (1.8–4.0)
			Gladstone et al. 2021^30^ SCREEN-AF	General population based, recruited from GP practices Region: Canada, Germany Age: ≥ 75 years Inclusion: CHADS_2_ ≥ 2 Exclusion: implantable PM/ICD/ILR, contraindication for OAC Known AF: excluded	Sequence: continuous, 14 days Biodata: single lead ECG Device: ZioPatch	80 ± 4	57%	434	5.3 (3.4–8)
			Kemp Gudmundsdottir et al. 2020^31^ STROKESTOP II High-risk subgroup	General population based Region: Stockholm Age: 75 or 76 years old Inclusion: NT-proBNP ≥ 125 ng/L Exclusion: -Known AF: excluded	Sequence: continuous, 14 days Biodata: single lead ECG Device: Novacor R-test 4 evolution	N/A	60%	3766	4.4 (3.8–5.1)
			Rooney et al. 2019^32^ ARIC	Community based Region: USA Age: ≥ 75 years Inclusion: Exclusion: implantable PM/ICD/ILR, skin allergic Known AF: separated	Sequence: continuous, 14 days Biodata: single lead ECG Device: ZioPatch	79.2 ± 4.6	58%	2244	2.5 (1.9–3.2)
			Steinhubl et al. 2018^33^ mSToPS	General population based (all subscribers of a certain health care plan9 Region: USA Age: ≥ 75 years or ≥ 65 years + Female + 1 comorbidity or ≥ 55 years + Male + 1 comorbidity Inclusion: -Exclusion: implantable PM/ICD, on OAC, known AT, known Aflutter Known AF: excluded	Sequence: continuous, 2 × 14 days Biodata: single lead ECG Device: ZioPatch	73.7 ± 7.0	41%	1738	2.9 (2.2–3.8)
			Heckbert et al. 2018^34^ MESA (substudy)	General population based (6 communities) Region: USA Age: ≥ 18 years Inclusion: -Exclusion: skin allergy to tape/adhesives Known AF: excluded	Sequence: continuous, 14 days Biodata: single lead ECG Device: ZioPatch	75 ± 8	48%	804	4.0 (2.7–5.6)
		PPG	Perez et al. 2019^15^ Apple Heart Study *own analysis of participants aged ≥ 65 years*	General population based Region: USA Age: ≥ 18 years Inclusion: Apple Watch owner Exclusion: -Known AF: included	Sequence: continuous Biodata: PPG + ECG patch Device: Apple Watch	N/A	42%^c^	24 626	3.1 (2.9–3.3)^a^
			Guo et al. 2019^16^ Pre-MAFA II (Huawei Heart Study) *own analysis of participants aged ≥ 65 years*	General population based Region: China Age: ≥ 18 years Inclusion: Huawei smartphone owner Exclusion: -Known AF: included	Sequence: continuous Biodata: PPG Device: Huawei Watch or Honor Watch or Honor Band	N/A	13%^c^	3419	2.8 (2.3–3.4)
			Lubitz et al. 2021^20^ FITBIT Heart Study *Design paper*	General population based Region: USA Age: ≥ 22 years Inclusion: Fitbit device owner Exclusion: on OAC, implanted PM/ICD Known AF: excluded (AF/AFlutter)	Sequence: continuous Biodata: PPG + ECG patch Device: Fitbit Ionic, Charge 3, Charge 4, Versa, Versa Lite, Versa 2, Versa 3, Sense, Inspire HR, Inspire 2	Analysis ongoing/unpublished			
	intermittent monitoring	single lead ECG	Berge et al. 2018^35^ ACE1950 Follow-Up	Community based Region: Sweden Age: 65 years Inclusion: ≥ 1 of HT, DM, HF, ST/TIA, VD Exclusion: -Known AF: excluded	Sequence: intermittent, 2 × daily, 14 days Biodata: single lead ECG Device: Zenicor	65	44%	1510	0.9 (0.5–1.5)
			Ghazal et al. 2020^36^	Community based Region: Stockholm regional, Sweden Age: ≥ 65 years Inclusion: -Exclusion: -Known AF: excluded	Sequence: intermittent, 3 × daily, 14 days Biodata: single lead ECG Device: Zenicor	72.1 (68.7, 75.9)^b^	61%	1010	2.7 (1.8–3.9)
			Halcox et al. 2017^37^ REHEARSE-AF	Community based, recruited from GP practices Region: Aberdeen region, UK Age: ≥ 65 Inclusion: CHA_2_DS_2_-VASc ≥ 2 (non-sex) Exclusion: on OAC, known CI for OAC, implanted PM/ICD Known AF: excluded	Sequence: intermittent, 2 × weekly, 1 year Biodata: single lead ECG Device: AliveCor	72.6 ± 5.4	52%	500	3.4 (2.0–5.4)
			Svennberg et al. 2015^38^ STROKESTOP (screening results)	General population based Region: Stockholm + Halland county, Sweden Age: 75 or 76 years old Inclusion: -Exclusion: -Known AF: separate	Sequence: intermittent, 2 × daily, 14 days Biodata: single lead ECG Device: Zenicor	N/A	55%	6507	3.8 (3.3–4.3)
	once	single lead ECG	Poulsen et al. 2022^39^	Community based, at preventive home visits Region: 3 Danish municipals Age: ≥ 65 years Inclusion: understanding Danish Exclusion: on OAC, home care or living in nursing home Known AF: excluded	Sequence: once per patient (2 repeated measures) Biodata: single lead ECG Device: Zenicor	80.9 (4.7)	56%	477	1.5 (0.6–3)
			Senoo et al. 2022^40^	Community based, at AF awareness campaign symposiums Region: Kyoto, Japan Age: ≥ 65 years Inclusion: -Exclusion: -Known AF: separate	Sequence: once per patient, 30 s Biodata: single lead ECG Device: Complete, Omron, Kyoto	72.4 ± 5.8	52%	1607	0.9 (0.5–1.5)
Systematic, GP-/Pharmacy-based, stationary	Once, cross-sectional	single lead ECG	Chen et al. 2020^41^ AF-CATCH, pre-phase	Community based, community health centres Region: Shanghai, China Age: ≥ 65 years Inclusion: -Exclusion: -Known AF: separate	Sequence: once per patient, 30 s Biodata: single lead ECG Device: AliveCor	71.6 ± 6.3	56%	4370	0.5 (0.3–0.8)
			Zaprutko et al. 2020^42^	General population based, recruited from 10 pharmacies Region: 6 cities in 3 regions of Poland Age: ≥ 65 years Inclusion: -Exclusion: -Known AF: excluded	Sequence: once per patient, 30 s Biodata: single lead ECG Device: AliveCor	73.7 ± 6.5	68%	525	1.4 (0.6–2.8)
			Orchard et al. 2020^43^ AF SMART II	Community based, recruited at GP practices Region: rural Australia Age: ≥ 65 years Inclusion: -Exclusion: implantable PM/ICD, on OAC Known AF: excluded	Sequence: once per patient, 30 s Biodata: single lead ECG Device: AliveCor	75.1 ± 6.8	53%	3103	1.2 (0.8–1.7)
			Gwynn et al. 2020^44^ *own analysis of participants aged ≥ 65 years*	Community based at 16 Aboriginal Community Health Organizations Region: rural Australia Age: ≥ 45 years Inclusion: Aboriginal heritage Exclusion: -Known AF: separated	Sequence: once per patient, 30 s Biodata: single lead ECG Device: AliveCor	N/A	56%	146	0.6 (0.0–3.7)
			Sun et al. 2022^45^ Hong Kong Outpatient AF screening study	Outpatient clinics (2 cardiology, 2 IM, 1 geriatric) Region: Hong Kong region Age: ≥ 65 years Inclusion: -Exclusion: dementia, terminal illness, unable to understand informed consent Known AF: separated	Sequence: once per patient, 30 s Biodata: single lead ECG Device: AliveCor	76.4 ± 7.8	49%	9734	3.0 (2.7–3.4)
		combination	Verbiest-van Gurp et al. 2022^46^	Outpatient clinics (2 cardiology, 2 IM, 1 geriatric) Region: Hong Kong region Age: ≥ 65 years Inclusion: Exclusion: known AF Known AF: separated	Sequence: once per patient Biodata: single lead ECG/BP/palpation Device: MyDiagnostick, Microlife BP Monitor, Radial pulse palpation	73.5 ± 5.5	54%	4339	0.8 (0.6–1.1)
		BP monitor	Jatau et al. 2022^47^ What’s Your Beat?	General population based, health centers, recruitment via media campaigns Region: Tasmania, Australia Age: ≥ 65 years Inclusion:—Exclusion: severe dementia, known cardiac arrhythmia, implantable PM/ICD Known AF: excluded	Sequence: once per patient Biodata: BP monitor Device: Microlife	71.0 (68.0–76.0)	59%	1704	0.9 (0.5–1.5)
	Intermittent	BP monitor	Denas et al. 2020^48^	General population based, GP practices Region: Veneto, Italy Age: ≥ 65 years Inclusion: -Exclusion: -Known AF: excluded	Sequence: 3 consecutive measurements per visit, unsystematically intermittent at visits over in average 410 days per patient Biodata: BP monitor Device: Microlife	75.5 ± 7.0	58%	14 987	2.5 (2.3–2.8)
		single lead ECG	Zhang et al. 2021^49^ AF-CATCH Quarterly-screened subgroup	Community based, community health centers Region: Shanghai Age: ≥ 65 years Inclusion: -Exclusion: -Known AF: excluded	Sequence: intermittent, quarterly Biodata: single lead ECG Device: AliveCor	71.3 ± 6.1	56%	2841	1.4 (1.0–1.9)
			Lubitz et al. 2022^50^ VITAL-AF	Community based at GP practices Region: Massachusetts, USA Age: ≥ 65 years Inclusion: -Exclusion: -Known AF: excluded	Sequence: intermittent over 12 months at GP visits Biodata: single lead ECG Device: AliveCor	73.9 ± 6.8	60%	15 393	1.7 (1.5–1.9)
		combination	Watanabe et al. 2022^51^ SCAN-AF	Hospital-affiliated outpatient clinics Region: Japan Age: ≥ 65 years Inclusion: CHA2DS2-VASc ≥ 2 or CHADS2 ≥ 1 Exclusion: known AF, use of AAD, inability to use the monitoring devices Known AF: excluded	Sequence: intermittent over 24 weeks Biodata: BP oscillogram, single lead ECG Device: Omron; myBeat	74.0 (69.0–79.0)	50%	1148	0.8 (0.4–1.5)
Cardiology patients without history of AF	continuous	Single lead ECG (ILR)	Svendsen et al. 2021^6^ LOOP	Cardiology patients, 4 centers Region: Denmark Age: 70–90 years Inclusion: ≥ 1 of HT, DM, ST/TIA, HF Exclusion: on OAC, known CI for OAC, implanted PM/ICD Known AF: excluded	Sequence: continuous, 64.5 months (59.3, 69.8)^b^Biodata: single lead ECG (ILR) Device: Medtronic Reveal LINQ ILR	74.7 ± 4.1	47%	1501	31.8 (29.0–34.8)
			Philippsen et al. 2017^52^	Diabetes and cardiology outpatient clinics at the Hospital of Southern Jutland Region: USA Age: ≥ 65 years Inclusion: HT (treated with ≥ 2 antihypertensives) and DM (treated with any antidiabetics or insulin) Exclusion: on OAC, EF < 45%, valvular disease needing intervention, implanted PM/ICD, known ischemic heart disease, ST/TIA, PAD, thyrotoxicosis, end-stage renal failure, severe obesity Known AF: excluded	Sequence: continuous, until ERI Biodata: PPG (ILR) Device: Medtronic ILR (Reveal XT or Reveal LINQ)	71.3 (67.4, 75.1)^b^	37%	82	20.7 (12.0–33.2)

The table lists the name of the study, the population studied, the design, age, and sex of the population, the number of participants, and the incidence rate of atrial arrhythmias with a 95% confidence interval. When age subgroup data was available or calculable, the study was included and parameters are reported for the population aged ≥ 65 years. Two studies are included of which only the design has been published (Lubitz et al.) or reported on a clinical trials website (AMALFI). Two implantable loop recorder studies are added at the bottom of the table.

**Abbreviations:** AAD, antiarrhythmic drug(s); BP, blood pressure; 95% CI, 95% confidence interval; CI, contraindication; CVD, any cardiovascular disease; DDys, diastolic dysfunction in echocardiography; DM, diabetes mellitus; EF, ejection fraction; GP, general practitioner; HF, heart failure; HT, hypertension; ICD, implantable cardioverter-defibrillator; ILR, implantable loop recorder; IM, internal medicine; IR, Incidence rate; IR/100 000, Incidence rate per 100 000 screened; n, Population size used for incidence rate calculation; OAC, oral anticoagulation; OSAS, obstructive sleep apnea syndrome; PAD, peripheral artery disease; PM, pacemaker; poAF, postoperative AF; ST/TIA, stroke or TIA; VD, vascular disease (in CHA2DS2-VASc).

a This study included participants without knowledge of the history of atrial fibrillation but was included in the review due to its sample size and population-wide approach. Reported is the incidence rate of screen-positive participants (irregular pulse notification). Of these, 34% were diagnosed on a subsequent Holter ECG.

b Median age and interquartile range (IQR) are reported.

c Proportion of females not available for reported age group.

Published studies in populations and cohorts including a subgroup with a comparable age range and mostly comparable screening technologies reported atrial arrhythmia detection rates between 2.8 and 3.1%,^15,16^ less than the smartphone and wearable PPG-based incidence rate in Smart in OAC—AFNET 9 of 5% in 4 weeks. When screened populations were enriched using clinical risk factors or elevated NT-proBNP concentrations,^31^ incidence rates increased (2.7–4.4%,^29,31,33^) Published reports suggest that continuous PPG monitoring is associated with higher (2.5 -5.3%,^30,32,34^) arrhythmia detection rates than intermittent monitoring (0.9–3.8%,^35–38^) confirmed in this study. The rate of ECG-confirmed AF in the Smart in OAC substudy via Holter or the clinical setting was of 3.1–3.4% of the overall study population, within the range we had estimated in this age group, but lower than previous PPG-detected arrhythmias in the same study, pointing to the paroxysmal character of AF. However, the confirmation rate of 60% (27 AF-positive out of 45 AA-positively screened participants) is higher than in the younger population of the Apple Heart Study.^15^ This has several potential explanations. One reason could be that Smart in OAC only screened for arrhythmia episodes lasting 6 min or longer, while Apple Heart accepted shorter arrhythmia durations. Three remotely conducted, large and population- and consumer-technology based landmark trials in AF screening via continuous PPG monitoring are the Apple Heart Study,^15^ the Pre-MAFA II trial (Huawei Heart Study)^16^ and the Fitbit Heart Study.^20^ The AA screen positive rates were 0.52%, 0.23%, and 1.0% in the overall screened population and 3.1, 2.8, and 3.6% in those aged 65 years and older.

Much higher detection rates were observed when opportunistic screening was performed or when data from implantable loop recorders were used to screen pre-selected, multimorbid patient populations.^6,52^ Subclinical AF episodes lasting longer than 6 min were detected in 26% of patients in a study of continuous single lead ECG monitoring.^34^ Studies employing implantable loop recorders also employed the cut-off of 6 min^6^ and a recent meta-analysis suggests that stroke risk is very low in patients with episodes shorter than 6 min.^9^

The AA detection yield in *Smart in OAC—AFNET 9* was slightly higher than in similar published screening trials in a comparable population only preselected by age above 65 years. Reasons for high AA yield could include the near continuous monitoring with a wearable PPG-sensor, and high compliance with wearing the device during nighttime. In participants in which AA was detected, the yield was nearly 1.6-fold elevated during nighttime (10 p.m to 6 a.m) compared with daytime even after correcting for better signal quality at night. In line with this observation, Deguchi et al.^53^ reported an elevated probability of AF onset around midnight from Holter-monitoring data of 217 patients with paroxysmal AF.

In 83% of participants, AA was detected within the first 28 days of monitoring and in most participants AA was detected within the first 14 days.

The minimal duration for arrhythmia detection used in Smart in OAC—AFNET 9 was 6 min.^21^ This is longer than the ESC guidelines definition of AF when detected on a clinical ECG, and longer than the minimal arrhythmia duration suggested for AF screening using consumer electronics in a recent EHRA guide.^7^ Rare arrhythmias of 6 min duration or more, detected within three months of screening using an implanted device, are associated with an increased risk of stroke.^10,22^ Six minutes of atrial arrhythmias are also sufficiently long to allow good differentiation of atrial arrhythmias from artefacts or other rhythm irregularities in wearables.^19^ These considerations informed our decision to screen for atrial arrhythmias of 6 min duration or more. The authors expect that there will be a gradual increase in the risk of ischaemic events that are preventable by oral anticoagulation as the arrhythmia duration, and by inference the arrhythmia burden, increases,^10^ a concept that was also presented in the most recent AFNET/EHRA consensus statement.^12^

It is therefore worth considering that screening pathways should address large cohorts or populations with rather short (14–28 days) but continuous monitoring periods, emphasizing night time monitoring, rather than unselective screening of smaller populations over a long time. Digital recruitment and consenting processes as demonstrated in *Smart in OAC—AFNET 9* can help include large populations even during a pandemic. The recently published eBRAVE-AF screening trial invited 67.488 German private healthcare policyholders aged 50 years and over of whom 5551 (8.2%) with a median age of 65 years were digitally enrolled.^54^ The study compared the use of a smartphone camera PPG-based intermittent screening application to usual care in a cross-over design and could show increased yields of newly diagnosed AF; additionally, the median age was older than the digitally enrolling Apple or Huawei Heart studies. *Smart in OAC—AFNET 9* targeted and enrolled an even older population following and openly advertised invitations independent of their insurer.

Apart from age, pre-selection of participants did not contribute to the increased screening yield in *Smart in OAC—AFNET 9*. Both age and self-reported estimated CHA_2_DS_2_-VASc-Score were comparable to or even lower than in similar studies^29–31,33,34^ and most participants were recruited via targeted public media communications and not from hospital patient pools. The ability of night-time recordings may however have increased the yield.

*Limitations.* While the communication around the study and the options for participation were designed to enable inclusive participation, we cannot exclude some selection of participants that may have influenced the observed atrial arrhythmia detection rate, based on access to a personal smartphone and wireless internet access. Our study targeted the older European population, and the participants were therefore mostly white. Observations may differ in other ethnicities. The remote study design relied on self-reporting of pre-existing medical conditions like known AF, hypertension, or diabetes as well as demographic data by participants. This may have contributed to comparably low reported rates of concomitant medical conditions in this population and also in the screen-positive AA group. Self-reported numbers in this study for hypertension and diabetes were similar to those observed in STROKESTOP.^5^

The design of our study included subsequent Holter ECG event recorder assessment in participants with positive PPG AA to ensure that participants would be reassured or receive a diagnosis of AF and subsequent treatment as required. Due to the transient nature of paroxysmal atrial arrhythmias and the lack of a simultaneous PPG signal analysis together with the ECG (as the PPG analysis stopped after a positive screen), the assessment in this study does not provide valid information on diagnostic accuracy. We still report results of Holter ECGs as these can be expected in clinical practice if PPG is used for screening. Performing Holter-ECGs on negatively screened participants was limited by operational difficulties as some participants without relevant findings were less keen to undergo further tests and staff of centres were less motivated to provide access to the Holter-ECGs to negatively screened participants during COVID-19 waves. In the future, this could be partially overcome by a central distribution system.

Adverse events directly associated with the PPG recording were minor skin reactions to the wristband and were only reported during the summer months (see Supplementary material online, *Table S1*). A changeable cotton wristband was offered to replace the standard silicone wristband and participants were able to use any personal wristband of their choice if it could be attached to the PPG unit.

Data on the cost effectiveness of the tested screening system have been published.^55,56^ The results of Smart in OAC—AFNET 9 will be an important component of a planned health economic (HE) analysis which is beyond the scope of this report.

## Conclusions

A fully digital, wearable based PPG screening identifies atrial arrhythmias in 5% of an openly invited population of older adults of 65 years or above without previously known AF or anticoagulation therapy. Advertising targeting older populations and remote technical support when needed enable broad participation and adequate monitoring durations. The majority of atrial arrhythmias were detected a few weeks after the initiation of screening.

SMART in OAC—AFNET 9 results provide robust information on the prevalence of PPG-detected atrial arrhythmias in older adults. The study provides data on different methods to reach out to such populations to offer arrhythmia screening and on patient characteristics with PPG-detected arrhythmias. The study thus generates robust information for the planning of an outcome trial.

## Author contributions

A.Z., E.G., L.F., R.B.S., T.H., P.K., and U.S. planned the study. R.B.S., P.K., L.F., A.Z.-.H., D.H., E.C., D.C., and K.J. designed the protocol. K.J., E.C., A.Z.-H., L.F., R.B.S., D.C., and E.G. designed lay-friendly information and prepared ethics applications. R.B.S., S.J.W., A.Z.-H., D.D., and E.G. contributed to participant recruitment and further diagnostics via study centres. A.Z. and E.V. were responsible for statistical analysis. J.O., K.J., and L.F. prepared the literature meta-analysis. E.V., J.O., and L.F. prepared result figures and tables. L.F., P.K., and E.V. wrote the manuscript. All authors made critical comments on the manuscript. L.F., E.V., J.O., P.K., and K.J. revised the study in response to reviewer comments.

## Supplementary Material

ztac067_Supplementary_Data

## Data Availability

Data will be made available by AFNET on reasonable request.

## Lead author biography

Prof Dr Larissa Fabritz studied medicine in Heidelberg, Münster, Lille, Concepcion, and Zurich, including a research year in Washington, DC, USA. Larissa trained at University Hospital Münster, Germany, with habilitation (D.S.) and specialist registration. In 2011, Larissa accepted a tenured clinical academic position at the University of Birmingham, UK, and was promoted to Chair in Cardiovascular Sciences there. She holds a Chair in Inherited Cardiac Conditions at the Center of Cardiovascular Sciences, UKE Hamburg, Germany. Larissa published on arrhythmias and cardiomyopathies. As a member of ESC working groups, EHRA, DZHK, and AFNET steering committee, she contributes to European research consortia and ESC guidelines.
