# Supplementary material for: Smartphone and wearable detected atrial arrhythmias in Older Adults: Results of a fully digital European Case finding study
Source: Eur Heart J Digit Health. 2022 Nov 1;3(4):610–25. doi: 10.1093/ehjdh/ztac067 (PMC9779806; doi:10.1093/ehjdh/ztac067)
Supplement: ztac067_Supplementary_Data [file ztac067_supplementary_data.docx]

# Data supplement

## Supplementary Table 1: Adverse events of interest. Skin irritations by the silicon wristband occurred only during the summer and were resolved by supplying a cotton wristband. These were the only adverse event related to the wearable. Adverse events are only listed as known.

| **Description of event** | P**articipants with event** |
| --- | --- |
| Skin irritation by wristband or Holter ECG | 16 |
| Death (cerebral insult) | 1 |
| Hospitalisation with atrial fibrillation and atrial flutter | 1 |
| Hospital attendance with chest pain, atrial fibrillation diagnosed | 1 |
| Unplanned cardiovascular hospitalisation due to aortic dissection and repair | 1 |
| Hospital attendance due to hypertension | 1 |

## Supplementary Table 2: Characteristics of the participants with and without confirmed AA at start of Holter ECG recording. ^1^ Fisher’s Exact Test for Count Data, ^2^ Linear Model ANOVA, ^3^ Trend test for ordinal variables

|  | **no AA (N=7)** | **confirmed AA (N=45)** | **Total (N=52)** | **p value** |
| --- | --- | --- | --- | --- |
| **Do you have heart failure (cardiac insufficiency, “a weak heart”) or reduced ejection fraction (reduced pumping ability of your heart)?** | 0 (0.0%) | 4 (8.9%) | 4 (7.7%) | 1.000^1^ |
| **Have you ever had a stroke (a blood clot clogging the vessels) a mini stroke (transient ischemic attack), or a disturbed blood flow in the brain?** | 0 (0.0%) | 2 (4.4%) | 2 (3.8%) | 1.000^1^ |
| **Have you ever had a heart attack?** | 0 (0.0%) | 2 (4.4%) | 2 (3.8%) | 1.000^1^ |
| **Have you ever had peripheral artery disease (so-called “window shopper’s disease”), aortic plaques or similar?** | 0 (0.0%) | 1 (2.2%) | 1 (1.9%) | 1.000^1^ |
| **ECG: Sex** |  |  |  | 0.033^1^ |
| Male | 0 (0.0%) | 21/44 (47.7%) | 21/51 (41.2%) |  |
| Female | 7 (100.0%) | 23/44 (52.3%) | 30/51 (58.8%) |  |
| **ECG: Hypertension** | 3 (42.9%) | 22 (48.9%) | 25 (48.1%) | 1.000^1^ |
| **ECG: Diabetes mellitus** | 1 (14.3%) | 0 (0.0%) | 1 (1.9%) | 0.135^1^ |
| **CHA_2_DS_2_VASc** |  |  |  | 0.864^2^ |
| Mean ± SD | 2.7 ± 0.8 | 2.6 ± 1.4 | 2.6 ± 1.3 |  |
| Median (Q1, Q3) | 3.0 (2.0, 3.0) | 2.0 (2.0, 3.0) | 2.0 (2.0, 3.0) |  |
| **Do you suffer from kidney disease(s)?** | 0 (0.0%) | 1 (2.2%) | 1 (1.9%) | 1.000^1^ |
| **ECG: EQ-5D: mobility** |  |  |  | 0.560^3^ |
| I have no problems in walking about | 6 (85.7%) | 40 (88.9%) | 46 (88.5%) |  |
| I have slight problems in walking about | 0 (0.0%) | 4 (8.9%) | 4 (7.7%) |  |
| I have moderate problems in walking about | 1 (14.3%) | 0 (0.0%) | 1 (1.9%) |  |
| I have severe problems in walking about | 0 (0.0%) | 1 (2.2%) | 1 (1.9%) |  |
| I am unable to walk about | 0 (0.0%) | 0 (0.0%) | 0 (0.0%) |  |
| **ECG: EQ-5D: self-care** |  |  |  | 0.693^3^ |
| I have no problems washing or dressing myself | 7 (100.0%) | 44 (97.8%) | 51 (98.1%) |  |
| I have slight problems washing or dressing myself | 0 (0.0%) | 0 (0.0%) | 0 (0.0%) |  |
| I have moderate problems washing or dressing myself | 0 (0.0%) | 1 (2.2%) | 1 (1.9%) |  |
| I have severe problems washing or dressing myself | 0 (0.0%) | 0 (0.0%) | 0 (0.0%) |  |
| I am unable to wash or dress myself | 0 (0.0%) | 0 (0.0%) | 0 (0.0%) |  |
| **ECG: EQ-5D: usual activities** |  |  |  | 0.444^3^ |
| I have no problems doing my usual activities | 7 (100.0%) | 41 (91.1%) | 48 (92.3%) |  |
| I have slight problems doing my usual activities | 0 (0.0%) | 3 (6.7%) | 3 (5.8%) |  |
| I have moderate problems doing my usual activities | 0 (0.0%) | 1 (2.2%) | 1 (1.9%) |  |
| I have severe problems doing my usual activities | 0 (0.0%) | 0 (0.0%) | 0 (0.0%) |  |
| I am unable to do my usual activities | 0 (0.0%) | 0 (0.0%) | 0 (0.0%) |  |
| **ECG: EQ-5D: Pain and discomfort** |  |  |  | 0.579^3^ |
| I have no pain or discomfort | 4 (57.1%) | 29 (64.4%) | 33 (63.5%) |  |
| I have slight pain or discomfort | 1 (14.3%) | 8 (17.8%) | 9 (17.3%) |  |
| I have moderate pain or discomfort | 2 (28.6%) | 8 (17.8%) | 10 (19.2%) |  |
| I have severe pain or discomfort | 0 (0.0%) | 0 (0.0%) | 0 (0.0%) |  |
| I have extreme pain or discomfort | 0 (0.0%) | 0 (0.0%) | 0 (0.0%) |  |
| **ECG: EQ-5D: anxiety and depression** |  |  |  | 0.389^3^ |
| I am not anxious or depressed | 7 (100.0%) | 40 (88.9%) | 47 (90.4%) |  |
| I am slightly anxious or depressed | 0 (0.0%) | 3 (6.7%) | 3 (5.8%) |  |
| I am moderately anxious or depressed | 0 (0.0%) | 2 (4.4%) | 2 (3.8%) |  |
| I am severely anxious or depressed | 0 (0.0%) | 0 (0.0%) | 0 (0.0%) |  |
| I am extremely anxious or depressed | 0 (0.0%) | 0 (0.0%) | 0 (0.0%) |  |
| **We would like to know how good or bad your health is TODAY. You will see a scale numbered from 0 to 100. 100 means the best health you can imagine. 0 means the worst health you can imagine. Please indicate on the scale how your health is TODAY. (eCRF)** |  |  |  | 0.519^2^ |
| Mean ± SD | 82.9 ± 14.1 | 78.8 ± 15.6 | 79.3 ± 15.4 |  |
| Median (Q1, Q3) | 85.0 (75.0, 92.5) | 80.0 (70.0, 90.0) | 80.0 (70.0, 90.0) |  |
| **ECG: EQ-5D 5L VT score** |  |  |  | 1.000^2^ |
| Mean ± SD | 0.95 ± 0.06 | 0.95 ± 0.07 | 0.95 ± 0.07 |  |
| Median (Q1, Q3) | 1.00 (0.90, 1.00) | 1.00 (0.89, 1.00) | 1.00 (0.89, 1.00) |  |
| **How many drinks containing alcohol do you have on average per week? (e.g. glass of wine, bottle of beer, shot of spirits or liquor)** |  |  |  | 0.736^1^ |
| Less than 1 | 3/4 (75.0%) | 16/32 (50.0%) | 19/36 (52.8%) |  |
| 1-5 drinks | 1/4 (25.0%) | 13/32 (40.6%) | 14/36 (38.9%) |  |
| 6-9 drinks | 0/4 (0.0%) | 2/32 (6.2%) | 2/36 (5.6%) |  |
| 10 or more | 0/4 (0.0%) | 1/32 (3.1%) | 1/36 (2.8%) |  |
| Prefer not to answer | 0/4 (0.0%) | 0/32 (0.0%) | 0/36 (0.0%) |  |
| **Do you smoke?** |  |  |  | 0.312^1^ |
| No, never-smoker | 2 (28.6%) | 26 (57.8%) | 28 (53.8%) |  |
| Yes, current smoker | 1 (14.3%) | 4 (8.9%) | 5 (9.6%) |  |
| No, but former smoker | 4 (57.1%) | 15 (33.3%) | 19 (36.5%) |  |
| **How user-friendly do you find the app/wearable?** |  |  |  | 0.359^1^ |
| Extremely user-friendly | 0 (0.0%) | 5 (11.1%) | 5 (9.6%) |  |
| Very user-friendly | 6 (85.7%) | 21 (46.7%) | 27 (51.9%) |  |
| Moderately user-friendly | 1 (14.3%) | 18 (40.0%) | 19 (36.5%) |  |
| Slightly user-friendly | 0 (0.0%) | 1 (2.2%) | 1 (1.9%) |  |
| Not at all user-friendly | 0 (0.0%) | 0 (0.0%) | 0 (0.0%) |  |
| **Did you experience any technical issues?** | 1 (14.3%) | 7 (15.6%) | 8 (15.4%) | 1.000^1^ |
| **Height [cm]** |  |  |  | 0.090^2^ |
| Mean ± SD | 164.1 ± 5.1 | 170.5 ± 9.5 | 169.7 ± 9.2 |  |
| Median (Q1, Q3) | 163.0 (161.0, 166.5) | 170.0 (165.0, 178.0) | 169.0 (163.0, 176.0) |  |
| **Weight [Kg]** |  |  |  | 0.115^2^ |
| Mean ± SD | 66.9 ± 9.1 | 79.2 ± 20.0 | 77.6 ± 19.3 |  |
| Median (Q1, Q3) | 68.0 (62.5, 71.5) | 78.0 (63.0, 90.0) | 75.0 (62.8, 90.0) |  |
| **Have you ever had a thyroid disease (e.g. hypothyroidism or hyperthyroidism)?** | 1 (14.3%) | 11 (24.4%) | 12 (23.1%) | 1.000^1^ |
| **Have you ever had cancer?** | 1 (14.3%) | 6 (13.3%) | 7 (13.5%) | 1.000^1^ |
| **Have you ever had a positive Covid-19 test?** | 2 (28.6%) | 2 (4.4%) | 4 (7.7%) | 0.083^1^ |
| **Ca antagonists** | 0 (0.0%) | 7 (15.6%) | 7 (13.5%) | 0.574^1^ |
| **Sartans** | 3 (42.9%) | 11 (24.4%) | 14 (26.9%) | 0.370^1^ |
| **Beta blocker** | 1 (14.3%) | 11 (24.4%) | 12 (23.1%) | 1.000^1^ |
| **Aspirin** | 0 (0.0%) | 2 (4.4%) | 2 (3.8%) | 1.000^1^ |
| **Diuretics** | 1 (14.3%) | 5 (11.1%) | 6 (11.5%) | 1.000^1^ |
| **Statins** | 0 (0.0%) | 4 (8.9%) | 4 (7.7%) | 1.000^1^ |
| **Other medics** | 0 (0.0%) | 2 (4.4%) | 2 (3.8%) | 1.000^1^ |
| **Have you ever been diagnosed with sleep apnea (i.e., breath stoppages while you sleep)?** |  |  |  | 1.000^1^ |
| No | 6 (85.7%) | 37 (82.2%) | 43 (82.7%) |  |
| Yes, it is treated with CPAP | 0 (0.0%) | 2 (4.4%) | 2 (3.8%) |  |
| Yes, it is not treated with CPAP | 1 (14.3%) | 6 (13.3%) | 7 (13.5%) |  |
| **Do you know about the disease atrial fibrillation?** | 3 (42.9%) | 33 (73.3%) | 36 (69.2%) | 0.182^1^ |
| **Are you worried about the results of the measurement?** |  |  |  | 0.731^1^ |
| No | 7 (100.0%) | 34 (75.6%) | 41 (78.8%) |  |
| Yes, a lot | 0 (0.0%) | 3 (6.7%) | 3 (5.8%) |  |
| Yes, a little | 0 (0.0%) | 8 (17.8%) | 8 (15.4%) |  |
| **Is the patient recommended to start treatment with NOAC, OAC, or other?** |  |  |  | 1.000^1^ |
| No | 0/0.0%) | 3/5 (60.0%) | 3/5 (60.0%) |  |
| Yes, NOAC | 0/0.0%) | 1/5 (20.0%) | 1/5 (20.0%) |  |
| Yes, OAC | 0/0.0%) | 1/5 (20.0%) | 1/5 (20.0%) |  |
| Yes, other | 0/0.0%) | 0/5 (0.0%) | 0/5 (0.0%) |  |
| **What is your highest education level?** |  |  |  | 0.283^1^ |
| No formal education | 0 (0.0%) | 2 (4.4%) | 2 (3.8%) |  |
| GCSE | 5 (71.4%) | 11 (24.4%) | 16 (30.8%) |  |
| A level | 0 (0.0%) | 10 (22.2%) | 10 (19.2%) |  |
| Undergraduate Degree | 0 (0.0%) | 1 (2.2%) | 1 (1.9%) |  |
| Master’s Degree | 0 (0.0%) | 9 (20.0%) | 9 (17.3%) |  |
| Doctorate | 0 (0.0%) | 1 (2.2%) | 1 (1.9%) |  |
| Prefer not to answer | 2 (28.6%) | 11 (24.4%) | 13 (25.0%) |  |
| **Have you recently experienced heart palpitations (i.e., irregular heartbeat, or the feeling that your heart is fluttering, skipping or racing)?** |  |  |  | 0.026^1^ |
| No | 5 (71.4%) | 15/44 (34.1%) | 20/51 (39.2%) |  |
| Yes | 1 (14.3%) | 28/44 (63.6%) | 29/51 (56.9%) |  |
| I don’t know | 1 (14.3%) | 1/44 (2.3%) | 2/51 (3.9%) |  |
| **Have you recently experienced fatigue (i.e., a feeling of constant physical and/or mental tiredness or weakness)?** |  |  |  | 0.721^1^ |
| No | 4 (57.1%) | 29/44 (65.9%) | 33/51 (64.7%) |  |
| Yes | 3 (42.9%) | 14/44 (31.8%) | 17/51 (33.3%) |  |
| I don’t know | 0 (0.0%) | 1/44 (2.3%) | 1/51 (2.0%) |  |
| **Have you recently experienced chest pain (a feeling like pressure or squeezing in your chest)?** |  |  |  | 0.662^1^ |
| No | 6 (85.7%) | 32/44 (72.7%) | 38/51 (74.5%) |  |
| Yes | 1 (14.3%) | 12/44 (27.3%) | 13/51 (25.5%) |  |
| I don’t know | 0 (0.0%) | 0/44 (0.0%) | 0/51 (0.0%) |  |
| **Have you recently experienced shortness of breath or difficulties in breathing?** |  |  |  | 0.685^1^ |
| No | 5 (71.4%) | 25/44 (56.8%) | 30/51 (58.8%) |  |
| Yes | 2 (28.6%) | 19/44 (43.2%) | 21/51 (41.2%) |  |
| I don’t know | 0 (0.0%) | 0/44 (0.0%) | 0/51 (0.0%) |  |
| **Have you recently felt dizzy or lightheaded?** |  |  |  | 0.657^1^ |
| No | 6 (85.7%) | 31/44 (70.5%) | 37/51 (72.5%) |  |
| Yes | 1 (14.3%) | 13/44 (29.5%) | 14/51 (27.5%) |  |
| I don’t know | 0 (0.0%) | 0/44 (0.0%) | 0/51 (0.0%) |  |
| **Have you recently experienced a syncope (i.e., fainting or passing out)?** |  |  |  | 0.137^1^ |
| No | 6 (85.7%) | 44/44 (100.0%) | 50/51 (98.0%) |  |
| Yes | 1 (14.3%) | 0/44 (0.0%) | 1/51 (2.0%) |  |
| I don’t know | 0 (0.0%) | 0/44 (0.0%) | 0/51 (0.0%) |  |

## Supplementary Figure 1. Literature review on wearable-detected atrial arrhythmias. The following search terms were used for a database search for the included systematic review of literature: Search 1 ((("Atrial Fibrillation" OR „Atrial Flutter" OR „atrial arrhythmia") AND ("Wearable Electronic Devices"[Mesh] OR "Mobile Applications"[Mesh] OR „Smartphone"[Mesh] OR "telemedicine" OR "mobile health" OR "mhealth")) NOT (Review[Publication Type] OR "Systematic Review"[Publication Type])); Search 2 ("Mass Screening"[MeSH] AND ("Atrial Fibrillation" OR „Atrial Flutter" OR „atrial arrhythmia") NOT (Review[Publication Type] OR "Systematic Review"[Publication Type] OR "Letter"[Publication Type] OR "Editorial"[Publication Type]))


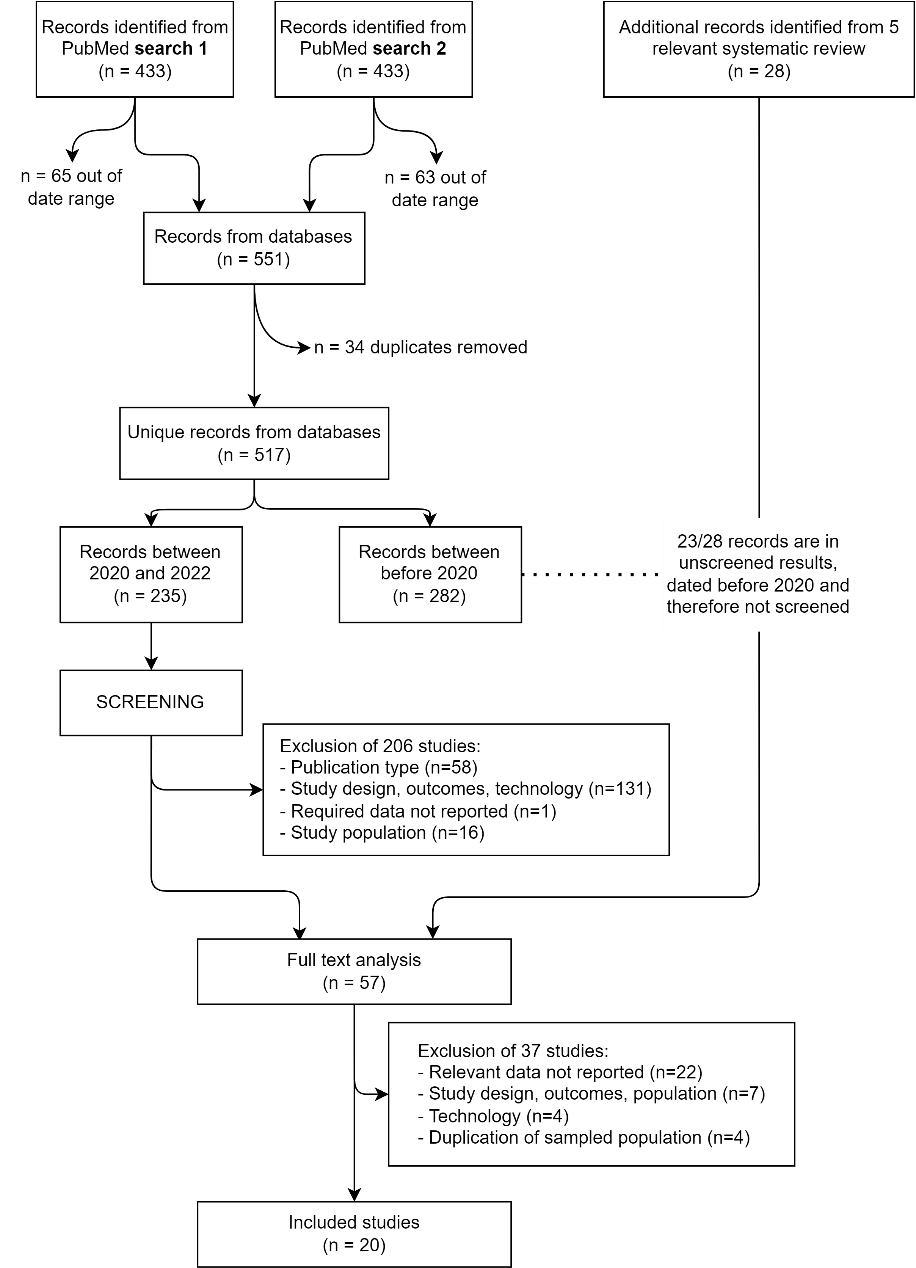


## Supplementary Figure 2. Enrolment and recruitment efforts (“other”) over time

**
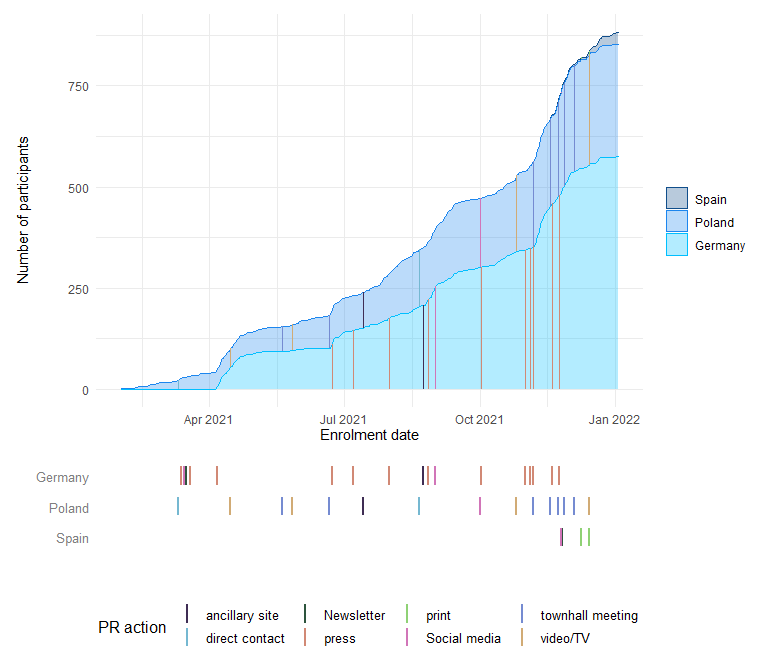
**
